# Equine Rhinitis A Virus Infection in Thoroughbred Racehorses—A Putative Role in Poor Performance?

**DOI:** 10.3390/v11100963

**Published:** 2019-10-18

**Authors:** Helena Back, John Weld, Cathal Walsh, Ann Cullinane

**Affiliations:** 1 Department of Virology, Immunology and Parasitology, National Veterinary Institute, SE-751-89 Uppsala, Sweden; Helena.Back@mpa.se; 2 Riverdown, Barrettstown, Newbridge, Co., Kildare W12HD83, Ireland; johnweld1@eircom.net; 3 Department of Mathematics and Statistics, University of Limerick, Castletroy, Limerick V94 T9PX, Ireland; Cathal.Walsh@ul.ie; 4 Virology Unit, The Irish Equine Centre, Johnstown, Naas, Co. Kildare W91RH93, Ireland

**Keywords:** equine rhinitis virus A, Thoroughbred racehorses, loss of performance

## Abstract

The aim of this study was to identify respiratory viruses circulating amongst elite racehorses in a training yard by serological testing of serial samples and to determine their impact on health status and ability to race. A six-month longitudinal study was conducted in 30 Thoroughbred racehorses (21 two-year-olds, five three-year-olds and four four-year-olds) during the Flat racing season. Sera were tested for the presence of antibodies against equine herpesvirus 1 and 4 (EHV-1 and EHV-4) and equine rhinitis viruses A and B (ERAV and ERBV) by complement fixation (CF) and equine arteritis virus (EAV) by ELISA. Antibodies against equine influenza (EI) were measured by haemagglutination inhibition (HI). Only ERAV was circulating in the yard throughout the six-month study period. Seroconversion to ERAV frequently correlated with clinical respiratory disease and was significantly associated with subsequent failure to race (*p* = 0.0009). Over 55% of the two-year-olds in the study seroconverted to ERAV in May and June. In contrast, only one seroconversion to ERAV was observed in the older horses. They remained free of any signs of respiratory disease and raced successfully throughout the study period. The importance of ERAV as a contributory factor in the interruption of training programmes for young horses may be underestimated.

## 1. Introduction

Equine respiratory infection together with lameness are the most common reasons for loss of training days and inability to race [1]. The main viruses associated with respiratory disease and loss of performance in racehorses are equine influenza virus (EI), equine herpesvirus 1 and 4 (EHV-1 and EHV-4) and equine rhinitis viruses A and B (ERAV and ERBV).

EI is a highly contagious virus and outbreaks of influenza may necessitate the cancellation of race meetings and other equestrian events [2]. However, in 1981, mandatory vaccination against EI was introduced for Thoroughbred racehorsesin Ireland and since then, no race meetings have been cancelled due to EI. However, outbreaks continue to occur in vaccinated horses, and young racehorses are particularly susceptible [3,4,5]. The return to athletic normality can be prolonged by damage to the mucociliary clearance mechanism and secondary bacterial infections [6].

EHV-1 and -4 circulate in horse populations worldwide and are associated with respiratory disease in horses, immunosuppression, reduced performance, abortion and occasionally, neurological disease [7,8,9]. Although there is no mandatory vaccination programme for EHV-1 and -4 in Ireland, some trainers vaccinate their horses in an effort to reduce the virus challenge in their yards.

ERAV and ERBV have also been identified worldwide in both healthy horses and horses with clinical respiratory signs [10,11,12,13,14,15,16]. A seroprevalence of 57% and 71% for ERAV and ERBV was recorded for Thoroughbred yearlings in Kentucky [17] and seroconversion to ERAV is common among young horses on entry to training yards [18,19,20]. Furthermore, ERBV was isolated in 30% of the horses with acute respiratory diseases in a Canadian survey [10].

There is a need for serological surveys to identify the viruses associated with economic loss in the racing industry. Longitudinal surveillance studies contribute to our understanding of the epidemiology of equine respiratory diseases in different countries and in different populations of horses. The aim of this serological study was to identify respiratory viruses circulating amongst elite racehorses of different ages in a training yard in Ireland and to determine their impact on health status and ability to race.

## 2. Materials and Methods

A six-month longitudinal study was conducted in 30 Thoroughbred racehorses during the Flat season. All the horses originated from the same stud farm and were in training with the same trainer. There were three different age groups of horses; the main group, i.e., 21 (70%) horses were two years of age, five (17%) of the horses were three years old and four (13%) were four years old. The two-year-old horses were in training for their first season. All the horses had been vaccinated against EI and EHV1 prior to entering the training yard.

At the request of the owner, whole blood samples were collected by the veterinary surgeon at monthly intervals from April to September. These were for routine serological screening and archiving as the “acute” samples for the testing of paired sera (“acute” and “convalescent”) in the event of an outbreak of respiratory disease. Additional blood samples were collected from horses with respiratory disease in mid-May.

Similarly to previous longitudinal studies of Thoroughbred racehorses in training [21,22,23], sera were tested for presence of antibodies against ERAV, ERBV, EHV-1 and EHV-4 using the Complement fixation (CF) test (Table S1). The test was performed as described by Thomson et al. [24], using guinea pig complement and sensitized sheep erythrocytes. Serial twofold dilutions of sera from 1:5 to 1:640 were tested. Sera were tested for antibodies against EI H7N7 and H3N8 viruses using the Haemagglutination Inhibition (HI) test. The sera were pretreated with potassium periodate, inactivated for 30 min at 56 °C (±1 °C) and tested as described previously [25]. Seroconversion was defined as a 4-fold or higher rise in CF or HI antibody titers. Sera were tested for the presence of antibodies against equine arteritis virus (EAV) by indirect ELISA (ID Screen^®^ Equine Viral Arteritis Indirect -Grabels, France).

A Chi-squared test was used to test the association of seroconversion with subsequent failure to race. The data was summarised in 2 × 2 contingency tables and an analysis was conducted in the R statistical software package version 3.5.1. The statistical significance was set at α = 0.05.

## 3. Results

The numbers of samples taken during the study period were distributed over the age groups as follows: 154 (71%) were from two-year-old horses, 35 (16%) were from the three-year-old horses and 28 (13%) of the samples were collected from four-year-old horses.

The two-year-old horses entered the training yard where the older horses resided in the beginning of April. At the time of first sampling, all the horses tested by HI (*n* = 26) had antibody titres against EI that were consistent with vaccination. All the horses had undetectable or low antibody titres (≤20) against EHV1 and 4. During the study period, five horses seroconverted to EI and 12 to EHV-1 and/or EHV-4 in response to vaccination. The remaining horses were not vaccinated against these viruses during the study period and no other seroconversions to EI, EHV-1 or EHV-4 were detected. All the horses were seronegative for EAV throughout the study period. Thus, there was no serological evidence of natural exposure to these viruses during the study period.

With the exception of two of the two-year-old horses (antibody titres of 80 and 40) all the horses had undetectable or low antibody titres (≤20) against ERAV at the time of first sampling in April. This was also true for ERBV, with the exception of one two-year-old with an antibody titre of 40. The first seroconversion to ERAV was observed at the second sampling occasion in the beginning of May. One seronegative two-year-old colt had mounted a significant antibody response to ERAV (0 to 160) within four weeks of arriving in the training yard. He did not exhibit clinical respiratory signs but was described by the trainer as very slow at work. Within the following two weeks, clinical signs, including inappetence, dullness, nasal discharge, limb oedema, enlarged submandibular lymph nodes and occasional coughing, were observed in seven of the in-contact two-year-old horses. They were returned to the stud farm of origin to recuperate where blood samples were collected for serological testing. Four of the horses seroconverted to ERAV during the first two weeks in May, two were seropositive with stable titres, and one seroconverted at a later time point.

**Figure 1:**
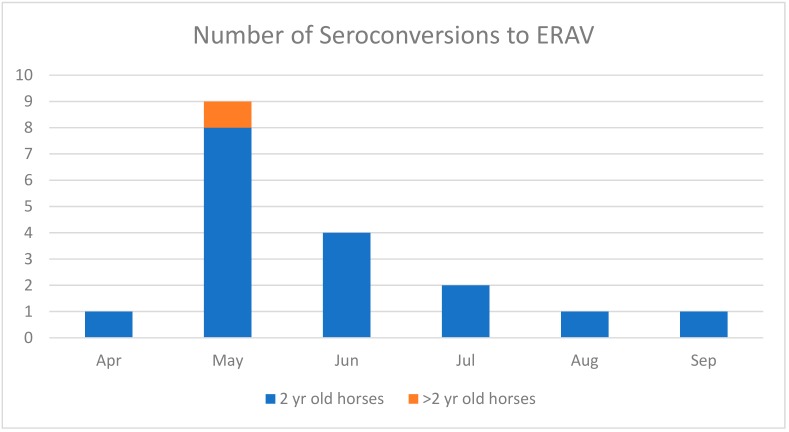
The number of horses that seroconverted to equine rhinitis A virus (ERAV) each month during the study period (April to September).

The ERAV serological results are summarized in Figure 1. Four additional seroconversions in two-year-old horses were identified by the end of May. Three of these horses had acute respiratory disease and were moved back to the stud farm. The fourth horse was subclinically infected and remained in training but was slow at work. By the end of June, four new seroconversions to ERAV were detected but only one horse had clinical respiratory signs and was returned home. One subclinically infected horse had previously seroconverted in early May.

The number of seroconversions to ERAV decreased with time, with two in July, one of which was a horse that had previously seroconverted in the first week of May. One horse seroconverted in August and one in September, which had seroconverted previously in May. In contrast to the initial exposure to virus, these seroconversions in autumn were not associated with clinical respiratory signs or loss of performance.

No seroconversions to ERAV or ERBV and no clinical respiratory signs were observed in the three-year-old horses during the study period. One four-year-old horse seroconverted to ERAV at the end of May, but no clinical signs were observed and he won a race five days before the seropositive blood sample was collected.

**Figure 2:**
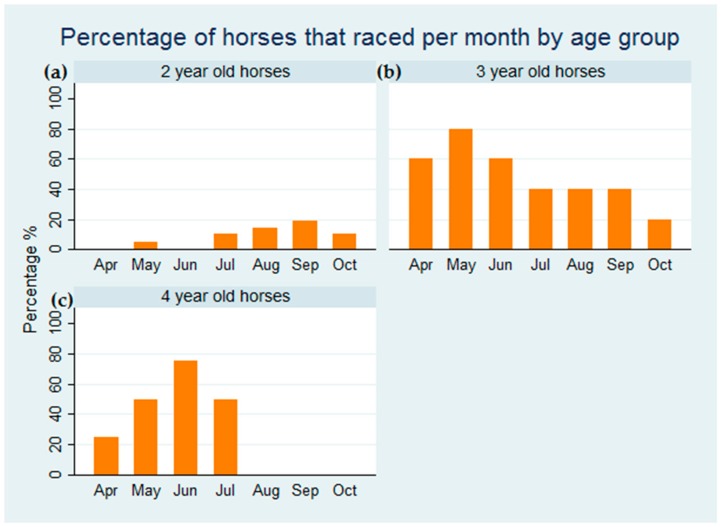
The percentage of horses that raced per month during the study period. The percentage of (**a**) two-year-old horses (**b**) three-year-old horses and (**c)** four-year-old horses that raced is shown.

In total, 18 seroconversions to ERAV were detected during the study period and 17 of them were in two-year-old horses. The majority, i.e. 13 (72%) of the seroconversions occurred in May and June. Only one of the two-year-old horses raced in May prior to being sent back to the stud farm to recover from respiratory disease. The two-year-old horses did not start racing after the respiratory episode until July, and, as can be seen in Figure 2, the percentage of two year-old-horses participating in race meetings was strikingly low when compared to the other two age groups. In total, during the study period, the five three-year-old horses raced 20 times and were placed first, second or third 12 times. The four older horses raced 12 times with six places. Only six of the 21 two-year-old horses raced. However, from 18 starts, they were placed 11 times. There was a significant association between seroconversion to ERAV and subsequent failure to race (*p* = 0.009).

The time from seroconversion to ERAV until the titers decreased to insignificant levels ranged from three weeks to five months, with a median value of two months and a mean value of 2.3 months. An association was observed between the antibody level and the rate of decline to original level.

Only one seroconversion to ERBV was observed in this study, a two-year-old horse seroconverted in June without any associated clinical respiratory signs.

## 4. Discussion

In this study, exposure to ERAV, as determined by serological testing, was associated with respiratory disease, loss of training days and failure to race in young racehorses. Monthly screening indicated that the majority of two-year-old horses were exposed to ERAV during the three months after entering the yard. The clinical signs that resulted in an interruption to training were primarily observed after the horses had been in the yard for several weeks and were being prepared for their first race. The susceptibility of young horses to rhinitis virus infection when moved to a new environment and starting to comingle with other horses has been reported previously [26,27]. In a seven-year serological study of racehorses in Japan, 69% of horses that seroconverted to ERAV were two years of age [16] and Black et al. [18] reported that 43% of horses seroconverted to ERAV within 7 months of entering a training stable in Australia. A previous study in a training yard in Ireland indicated that ERAV infection was largely confined to two-year-old horses and was most prevalent in late winter and spring [19]. However, we believe that this is the first investigation of the association between ERAV infection with interrupted training and failure to race. Over 55% of the two-year-olds in the study seroconverted to ERAV in May and June and none of them raced at that time. In fact, only 29% of the two-year-olds raced in their first season.

The results of this study suggest that the importance of ERAV in the interruption of training programmes for young horses may be underestimated. Acute febrile respiratory disease following ERAV infection has been reported previously [11,15,28,29]. In an experimental study, ERAV-inoculated ponies developed respiratory tract disease characterized by pyrexia, nasal discharge, adventitious lung sounds, and enlarged mandibular lymph nodes, which corresponded with an increase in antibody titres against the virus [29]. The clinical signs observed during this study were associated with the seroconversions and there was no evidence of other viral infections. ERAV may have been the primary cause of the respiratory disease and subsequent training loss, but it is more likely that the virus was a contributory factor in a multifactorial disorder. The serology tests used in this study are sensitive and specific, but it is possible that other less common viruses, such as adenovirus and coronavirus, or even as-yet unidentified equine viruses, may have played a role. Previous studies have demonstrated the complexity of poor performance syndrome in Thoroughbred racehorses and that bacteria are more common and may be aetiologically more important than viruses [21,22,23]. Unfortunately, the possible role of bacterial infection as a cofactor was not investigated in this study and the statistically significant association of ERAV with failure to race during the study period does not conclusively demonstrate causation. However, it is essential to determine which infectious agents are prevalent in populations that suffer interruption to training as a first step in assessing their true impact.

Lack of previous exposure to ERAV and stress associated with the change of environment and intensive training may have contributed to disease susceptibility. In contrast to the two-year-old horses, no seroconversions to ERAV were observed in the three-year-old horses—they remained free of any signs of respiratory disease and raced successfully throughout the study period. The seroconversion to ERAV in the four-year-old horse and the reinfection of two of the younger horses were not associated with clinical signs or reduced performance. Experimental studies have indicated that ERAV infection stimulated a protective response and that reinfection is asymptomatic [29].

The serology results indicate that ERAV was circulating in the yard throughout the six month study period. The dynamics of ERAV infection in horse populations are poorly understood but virus persists in horses even in the presence of high levels of antibodies [26,28]. ERAV can be isolated from the blood for a few days post infection and from the nasopharynx and faeces for up to a month [28]. Studies in the USA, Ireland and Australia detected ERAV in post-race urine samples at frequencies of 17%, 29% and 23%, respectively, which led to a suggestion that the persistent presence of ERAV in urine may contribute to its maintenance in training yards [17,30,31]. ERBV was not detected in urine [17,30]. During this study period, only one horse seroconverted to ERBV. This was consistent with an earlier study in Ireland in which ERAV was found to be more commonly detected than ERBV [30]. Co-circulation of ERAV and ERBV was also reported previously [30].

The low seroprevalence of ERAV and ERBV detected in the older horses at the beginning of this study may be due to the use of the CF test rather than the virus neutralization test (VNT) favored by some investigators [13,18,32]. The CF test is useful for the diagnosis of acute infections, as positive CF titres are often an indication of immunoglobulin M (IgM) antibodies or very high levels of IgG. Neutralizing antibodies which are mainly IgG persist for many years and the VNT is the test of choice for seroprevalence studies [33]. Burrows (1969) [34] reported that 59% of mares had neutralizing antibody against ERAV, in contrast to 10% of foals and yearlings, and suggested that most infection occurs during the period of training and racing. The relationship between neutralizing antibodies and clinical protection has been established Diaz-Mendez et al. [29]. It is likely that the older horses in this study that were seronegative by the CF test were exposed to ERAV in their first year of training and had neutralizing antibodies at the start of the study that were sufficient to protect them from infection and subsequent seroconversion, as measured by the CF test. However, this hypothesis remains unproven and it is also possible that although they were in the same yard as the two-year-old horses, they may not have been exposed to the virus. In this study, the CF antibodies declined to the original levels within months, confirming that they are not persistent and thus, a useful indicator of recent exposure to virus. As no relationship between CF antibodies and protection has been proposed, an investigation of an outbreak of ERAV including the measurement of neutralizing antibodies would give useful insight into protection.

In summary, this study demonstrates that routine serological monitoring of young racehorses by CF test revealed that ERAV is potentially an important contributor to training loss in young racehorses and merits further investigation with larger study populations and a more comprehensive testing regime. Unfortunately, only five nasopharyngeal samples were collected during this study, and all were negative for EHV1, EHV4, EI, EAV, ERAV and ERAB by real time PCR (data not shown). None of the nasal swabs were collected at the optimal time for detection of ERAV, which experimental infections suggest is from one to 12 days post-infection [31]. Further investigations in which samples are methodically collected for virus detection, serology and bacteriological investigation would be beneficial in elucidating the role of ERAV and other pathogens in the poor performance in young racehorses. However, all such field studies are dependent on the cooperation of the owner and the availability of horses.

ERAV is not contained in any of the vaccines that are currently available. As no clinical signs were attributable to reinfection, it is likely that immunization would be effective. The results of this study suggest that strategic vaccination of young horses against this virus could reduce economic loss due to respiratory disease and interruption of training schedules.

## Supplementary Materials

The following are available online at https://www.mdpi.com/1999-4915/11/10/963/s1.
