# Supplementary material for: Equine Rhinitis A Virus Infection in Thoroughbred Racehorses—A Putative Role in Poor Performance?
Source: Viruses. 2019 Oct 18;11(10):963. doi: 10.3390/v11100963 (PMC6848918; doi:10.3390/v11100963)
Supplement: Supplementary file 1 [file viruses-11-00963-s001.zip › viruses-602351-suppl/Legend Supplementary Table 1.pdf]

Supplementary Table 1

The table summarises the serology results. The samples were tested for antibodies against equine herpesvirus 1(EHV-1), equine herpesvirus 4 (EHV-4) equine rhinitis virus A (ERAV), equine rhinitis virus B (ERBV) using the complement test fixation (CFT), for antibodies against equine influenza (EI) of the H7N7 and H3N8 subtypes using the haemagglutination inhibition (HI) test and for antibodies against equine arteritis virus (EAV) using an indirect enzyme linked immunosorbent assay (ELISA). The seroconversions to ERAV are highlighted in blue. The seroconversions to EHV1, EHV4 and EI are indicated in red font. * indicates results for a single horse post vaccination against EHV1, that were considered to be post-vaccinal cross-reacting antibody to rhinitis virus antigens used in the CFT i.e. false positive seroconversions, and thus excluded from the analysis. N/A indicates not tested.
